# Inflammatory myofibroblastic tumor of the urinary bladder

**DOI:** 10.4103/0974-7796.65106

**Published:** 2010

**Authors:** Vipul Yagnik, Amit Chadha, Sanjay Chaudhari, Keyuri Patel

**Affiliations:** Department of Surgery Pramukhswami Medical College, Karamsad-388 325, Gujarat, India; 1 Department of Pathology, Pramukhswami Medical College, Karamsad-388 325, Gujarat, India

**Keywords:** Immunohistochemical staining, inflammatory myofibroblastic tumor, spindle myoepithelial cell proliferation

## Abstract

Inflammatory myofibroblastic tumor (IMT) of bladder is an uncommon benign tumor of bladder, which is of unknown neoplastic potential, characterized by spindle cell proliferation with characteristic fibroinflammatory and pseudosarcomatous appearance. Essential criteria for the diagnosis of IMT are: spindle myoepithelial cell proliferation and lymphocytic infiltrate. Complete surgical resection is the treatment of choice.

## INTRODUCTION

An inflammatory myofibroblastic tumor (IMT) is very rare in the genitourinary tract. We report here a case of IMT of the urinary bladder and discuss its clinical presentation, diagnosis, and management.

## CASE REPORT

**Figure 1:**
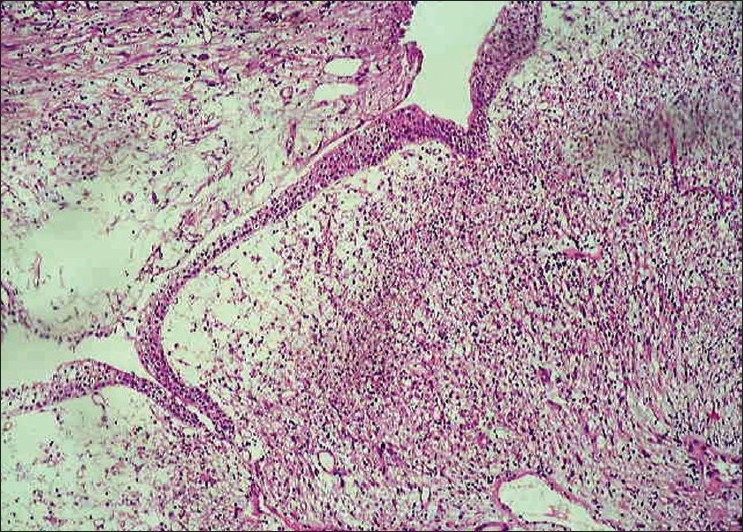
Urinary bladder epithelium (H and E, ×100 times)

**Figure 2:**
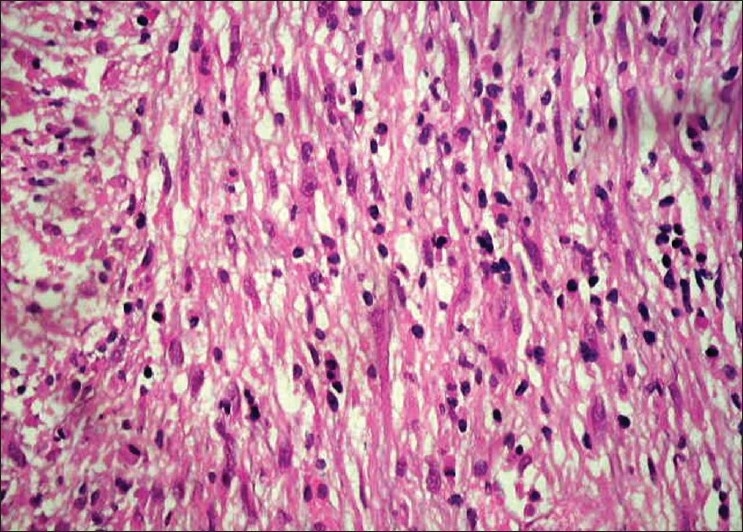
Spindle myoepithelial cell proliferation (H and E, ×400)

A 30-year old male in surgical outpatient department presented with a complaint of gross hematuria since previous one week. The patient complained of burning micturation and denied any history of fever, trauma, recurrent UTI, and sexually transmitted diseases. There was no past history of a similar attack or tuberculosis. The patient was investigated through CT abdomen, which showed irregular heterogeneously enhancing polypoidal bladder base lesion infiltrating bilateral seminal vesicle. X-ray chest was normal. Urine cytology was negative for malignant cell. All blood investigations including renal function test were normal, except hemoglobin which was 9 g/dl. The patient underwent cystoscopy, which showed large sessile smooth-walled growth arising from the bladder base and posterior wall in continuity with prostate. Multiple biopsies were taken from the tumor. Microscopically, tumor was composed of a few spindle cells and inflammatory cells comprised lymphocytes and plasma cells [Figures 1 and 2]. On immunohistochemistry, tumor expressed Desmin, SMA and ALK-1 and was immunonegative for cytokeratin and myogenin.

## DISCUSSION

An IMT of bladder is an uncommon benign tumor of bladder of unknown neoplastic potential characterized by spindle cell proliferation with characteristic fibroinflammatory and pseudosarcomatous appearance. It is also known as pseudosarcoma, atypical myfibroblastic tumor, atypical fibromyxoid tumor, plasma cell granuloma, etc.[1] It is idiopathic and no known predisposing condition exist for myofibroblastic tumor of the bladder.[2] The most common site for this tumor is lung.[3] It can affect any age group, but is more common in children and young adults with slight female preponderance (F:M ratio 3:4). It is rare in the genitourinary tract with the most common site being urinary bladder. The first case was reported by Roth in 1980.[4] Origin of IMT is controversial, but a recent report suggests that it is neoplastic because of its aggressive behavior, involvement of chromosome 2p23 and cytogenetic clonality. Essential criteria for the diagnosis of IMT are: spindle myoepithelial cell proliferation and lymphocytic infiltrate. Immunohistochemical staining may demonstrate positivity for anaplastic lymphoma kinase, vimentin, cytokeratin. Anaplastic lymphoma kinase (ALK) has been described as a good marker for IMT. Myogenin, a potent marker for rhabdomyosarcoma, helped in exclusion of this tumor.[5] Because of its highly cellular nature and aggressive behavior, it can be confused with malignancy. Initial biopsy and full histopathological examination is recommended where complete resection is problematic. Complete surgical resection is performed to avoid local recurrence.

To summarize, an IMT is a rare tumor of the urinary bladder. High index of suspicion is required for diagnosis. Biopsy is the gold standard for diagnosis and immunohistochemistry is very important to confirm the diagnosis. Surgical resection is the treatment of choice
